# First records of the genera *Histeromerus* Wesmael (Hymenoptera, Braconidae, Histeromerinae) and *Ecclitura* Kokujev (Hymenoptera, Braconidae, Euphorinae) in Italy

**DOI:** 10.3897/zookeys.310.5136

**Published:** 2013-06-19

**Authors:** Sergey A. Belokobylskij, Augusto Loni, Andrea Lucchi, Umberto Bernardo

**Affiliations:** 1 Zoological Institute Russian Academy of Sciences, St. Petersburg, 199034, Russia; Museum and Institute of Zoology, Polish Academy of Sciences, Wilcza 64, Warszawa 00–679, Poland; 2 Department of Agriculture, Food and Environment, Pisa University, Via del Borghetto, 80 56124 Pisa, Italys; 3 Istituto per la Protezione delle Piante, Consiglio Nazionale delle Ricerche, Sezione di Portici,Via Università, 133, Portici 80055, NA, Italy

**Keywords:** Braconidae, *Histeromerus*, *Ecclitura*, parasitoids, new records, redescriptions

## Abstract

Braconid genera *Histeromerus* Wesmael, 1838 from subfamily Histeromerinae and *Ecclitura* Kokujev, 1902 from subfamily Euphorinae are recorded in the fauna of Italy for the first time. The discussions about taxonomic position, morphological characters and composition of these genera as well as the redescriptions of the genus and species of *Ecclitura primoris* Kokujev are given.

## Introduction

Family Braconidae represents one of the largest and diversified families of parasitic Hymenoptera, including, with a few exceptions, mainly primary parasitoids. These occur in very diverse habitats and result linked to a wide range of hosts. As a fact, braconids can represent an interesting ecological resource as sensitive indicators of environmental richness and stability and of local diversity. The regulatory effect which they exert on host insects population derives from a very wide diversity of physiological and behavioural adaptations (Wharton 1993). On the other hand, the ecological information about many species of this family is scarce, so that any information regarding new findings in new areas is very welcome, as well as any information related to the habitat context where recordings are made. This paper deals with the first Italian record of *Histeromerus mystacinus* Wesmael and *Ecclitura primoris* Kokujev. Italian peninsula extends over a wide range of latitudes with numerous mountain ranges along its length. Such a geomorphological configuration results in many different environments suited to host a large insect biodiversity. Particularly interesting is the finding of *Ecclitura primoris* both in central (Tuscany) than southern (Campania) Italy, in two different environmental contexts. Nowadays, this species was considered very rare on the base of its reduced world distribution (Shaw 1997). Discovery of *Histeromerus mystacinus* in Italy is also very interesting because this is first record of this taxon on the real Mediterranean territory.

Terminology adopted for morphological features and measurements follows Belokobylskij and Maetô (2009). Wing venation nomenclature follows Belokobylskij and Tobias (1998) and Belokobylskij and Maetô (2009). The studied materials are kept in the collections of the Zoological Institute of the Russian Academy of Sciences (St. Petersburg, Russia) and Department of Agriculture, Food and Environment, Pisa University (Pisa, Italy).

## Taxonomic part

### Genus Histeromerus

Wesmael, 1838

http://species-id.net/wiki/Histeromerus

#### Type species.

*Histeromerus mystacinus* Wesmael, 1838

#### Comments.

*Histeromerus* Wesmael is a type genus of the monotypic subfamily Histeromerinae. The systematic position of this peculiar genus changed many times during its study. For a long time *Histeromerus* was considered as a member of subfamily Doryctinae and included in the tribe Doryctini or Histeromerini (Shenefelt and Marsh 1976, Yu et al. 2012), but other authors treated it inside the subfamily Braconinae (Achterberg 1976, Quicke 1987) or as a member of the separated subfamily Histeromerinae (Achterberg 1984, Quicke and Achterberg 1990, Belokobylskij 1998). According to the more recent molecular phylogenetic study on the cyclostome subfamilies (Zaldivar et al. 2006, Sharanowski et al. 2011), this genus was put inside subfamily Rhyssalinae. On the other side, numerous apomorphic characters of this taxon (prepectal carina and metapleural flange absent, fore and middle tibiae with clusters of spines, hind basitarsus about twice longer than remainder of hind tarsus, first metasomal tergite without sublateral grooves, dorsope absent, ovipositor compressed and without apical notch or teeth, etc.) did not show a close relation of *Histeromerus* with any rhyssaline taxa so seems better to keep this genus in the separate subfamily Histeromerinae until receiving of additional and more robust information about its taxonomic position.

Hitherto, four species of *Histeromerus* are known. Type species *Histeromerus mystacinus* was recorded only in the Western Palaearctic (including Iran). *Histeromerus canadensis* Ashmead, 1891, mainly known from U.S.A. and Canada, was also found in the Netherlands. *Histeromerus orientalis* Chou & Chou, 1991 was described from Taiwan and later was additionally recorded in Japan (Ogasawara Islands). Finally a fourth species, *Histeromerus clavatus* Austin & Wharton, 1992, was described from Australia (Queensland) (Yu et al. 2012).

*Histeromerus mystacinus* Wesmael is widely distributed in several European countries and also in Caucasus and Central Asia (North Iran). On the other hand, this species practically resulted unknown for the south part of Europe (Mediterranean coast). First discovery of *Histeromerus mystacinus* in Italy is very interesting, because expands the geographical distribution of this taxon in dry territories. Members of *Histeromerus* are gregarious ectoparasitoids of concealed living beetles’ larvae from subfamilies Anobiidae, Buprestidae, Cerambycidae, Cisidae, Elateridae, Lucanidae, Lyctidae, and Ptinidae (Yu et al. 2012).

### Histeromerus mystacinus

Wesmael, 1838

http://species-id.net/wiki/Histeromerus_mystacinus

Figures 1–9

#### Material examined.

Italy: 1 female, Campania, Torre del Greco, (NA), Parco del Vesuvio, 3–18.VII.2007, Guerrieri leg.

#### Distribution.

Ireland, U.K., Belgium, Netherland, Denmark, France, Germany, Czech Republic, Hungary, Italy (first record), Sweden, Poland, Slovakia, Ukraine, Bulgaria, Russia (European part), Georgia, Iran.

**Figures 1–9.**
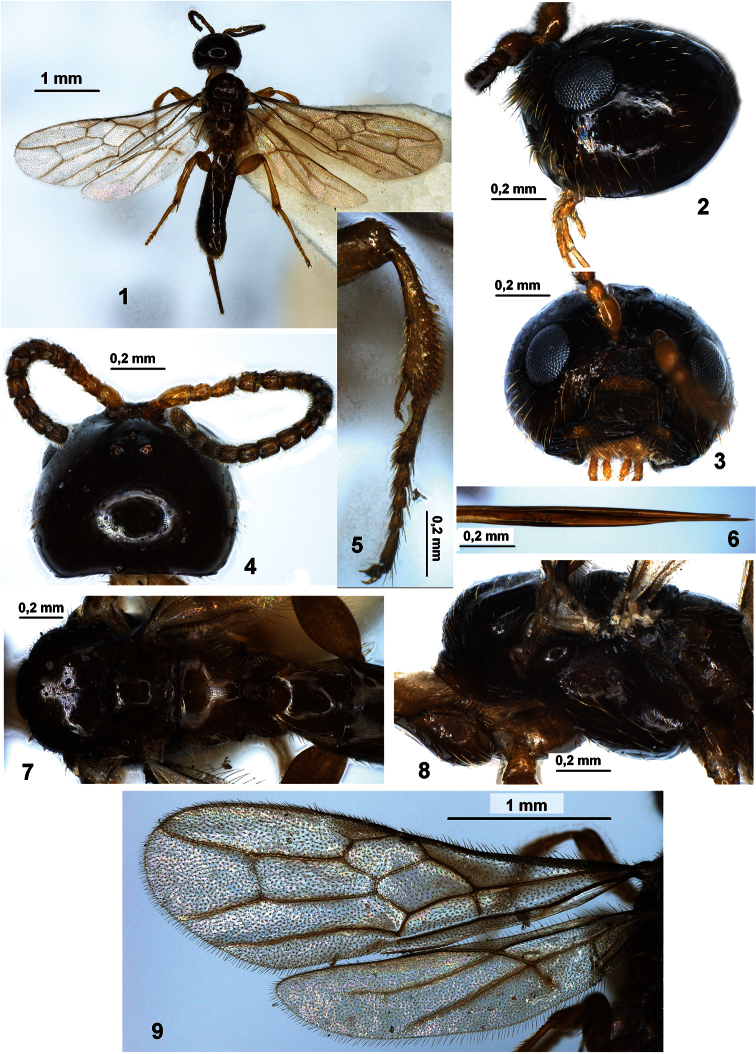
*Histeromerus mystacinus* Wesmael (female) **1** habitus, dorsal view **2** head, lateral view **3** head, front view **4** head and antenna, dorsal view **5** fore tibia and tarsus **6** tip of ovipositor **7** mesosoma and first metasomal tergite, dorsal view **8** mesosoma, lateral view **9** fore and hind wings.

### Genus Ecclitura

Kokujev, 1902

http://species-id.net/wiki/Ecclitura

#### Type species.

*Ecclitura primoris* Kokujev, 1902.

#### Comments.

*Ecclitura* Kokujev is until recently monotypic genus originally described from Turkmenistan in the beginning of 20th century (Kokujev 1902).

Taxonomic position of *Ecclitura* was discussed in several publications. Muesebeck (1936) defined this genus as the most similar to *Perilitus* Nees, 1819 and *Rhopalophorus* Curtis, 1837 on the base of the wings venation and the long exerted ovipositor. Tobias (1966) in his paper about generic groupings and evolution of subfamily Euphorinae included *Ecclitura* in the *Perilitus* group together with the genera *Perilitus*, *Microctonus* Wesmael, 1835, *Dinocampus* Foerster, 1862, *Streblocera* Westwood, 1833, *Ropalophorus* and *Centistina* Enderlein, 1912. Shaw (1985) in his phylogenetic study of subfamily Euphorinae, concluded that *Ecclitura* is a sister group of *Streblocera* focusing the synapomorphies on the lack of the first medial abscissa in the fore wing, the long and slender scape and the presence of dorsope on the first metasomal tergite. He included this genus in the tribe Microctonini (section 3) together with *Microctonus*, *Proclithrophorus* Tobias & Belokobylskij, 1981, and *Streblocera* and separated the tribes Perilitini (only with *Perilitus*) and Dinocampini (with *Centistina*, *Dinocampus* and *Rhopalophorus*). However, Tobias (1986) in his key to European species of Euphorinae retained the previous position of this genus in the tribe Perilitini. In this way he *de facto* synonymized Microctonini and Dinocampini with this tribe, but separated *Proclithrophorus* in the new tribe Proclithrophorini. Belokobylskij (2000), in the new key to Palaearctic euphorine genera, synonymized with Perilitini also tribes Cryptoxilonini and Townesilitini. *Ecclitura* here was related with *Streblocera* and *Heia* Chen & Achterberg.

Knowledge on molecular phylogeny and its implication for classification of subfamily Euphorinae are very limited. A specific publication for this task (Li et al. 2003) studied only a few taxa and a single gene (ribosomal 28S). This investigation included in the same group the genera *Microctonus* and *Streblocera* (and possibly related *Ecclitura*), but monophyly of the tribe Microctonini was not resolved. New preliminary information about Euphorinae phylogeny on the base of combined morphological (37 characters) and molecular (4 markers: 18S, 28S, CAD, COI) data was suggested by Stigenberg and Boring (Internet, unpublished data). One of the result of this work was the arrangement of *Ecclitura* in the tribe Dinocampini. A final and more comprehensive published information about this topic should help to better understand the real results of this vast and diverse investigation.

The single described species of this genus, *Ecclitura primoris*, was recorded in the Western Palaearctic Region. Two other undescribed species of this genus were reported also from U.S.A. (Shaw 1985), India and Vietnam (Belokobylskij 2000). The discovery of these genus and species in Italy makes it likely the finding of *Ecclitura* also in other climatically similar localities of South Europe and North Africa. No data are available about *Ecclitura* hosts. However, being phylogenetically related with the genus *Streblocera* (associated with Chrysomelidae) and sharing with this genus distinctive morphological features (structure of antenna, first metasomal tergite and ovipositor shapes: Shaw 1985, 2004) *Ecclitura* could be also imagobiont and parasitoid of the adults of some beetles (Chrysomelidae or Curculionidae).

Four females of *Ecclitura* were collected by Malaise traps closely to Napoli in the Campania Province. Fourteen females were captured in 2012 in three vineyards in the surroundings of Pisa (Tuscany), by using Malaise traps. These traps worked from the half of May to the half of October. *Ecclitura* specimens were captured from July 12 to October 4. Hitherto, no males of this species have never been recorded over area of his distribution, and it is possible *Ecclitura primoris* reproduces by thelytokous modality as already observed for several other species of Euphorinae (Shaw 2004, Reumer et al. 2012). We have redescribed below the genus *Ecclitura* as well as its type species, because their original descriptions were incomplete and because variability of morphological characters of these taxa were investigated on the base of additional material from different localities.

#### Redescription of the genus.

**Figures 10–16.**
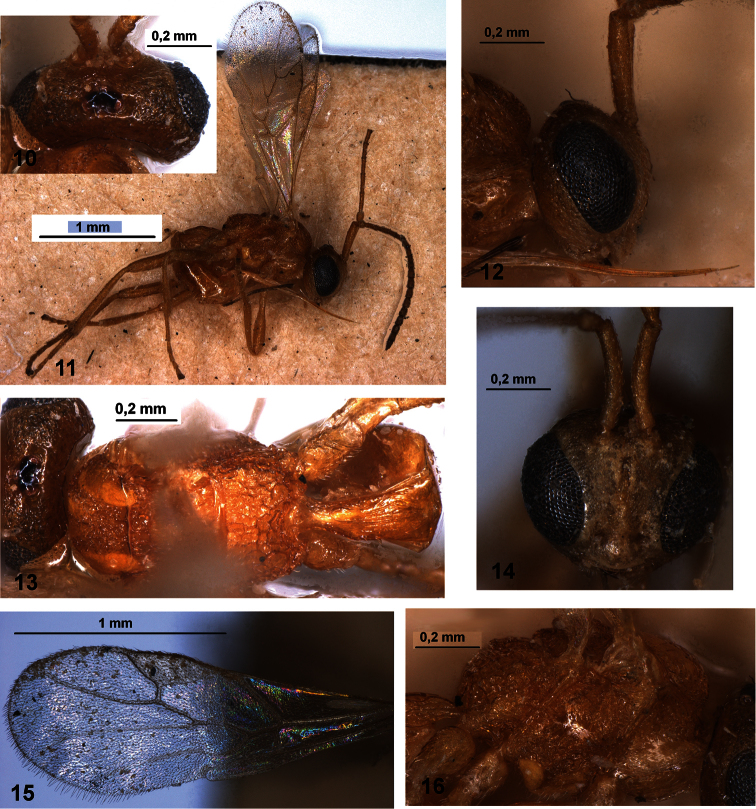
*Ecclitura primoris* Kokujev (holotype, female, Turkmenistan) **10** head, dorsal view **11** habitus, dorsal view **12** head and scape of antenna, lateral view **13** mesosoma and first metasomal tergite, dorsal view **14** head and scape of antenna, front view **15** fore wing **16** mesosoma, lateral view.

**Figures 17–27.**
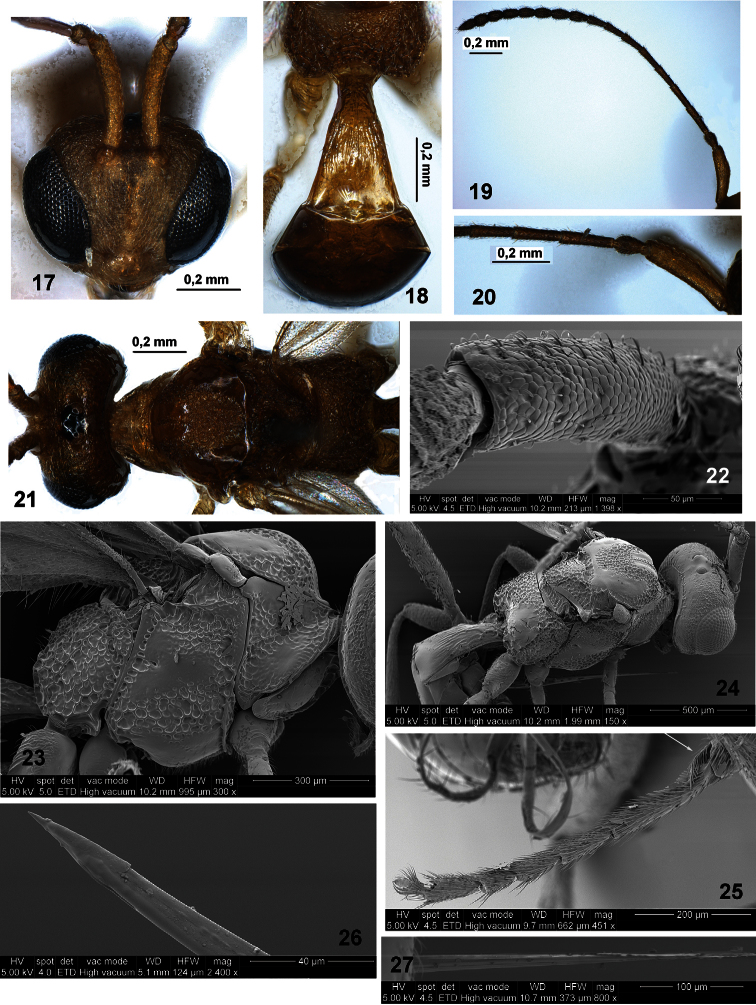
*Ecclitura primoris* Kokujev (female, Italy) **17** head and scape of antenna, front view **18** first metasomal tergite, dorsal view **19** antenna, lateral view **20** basal part of antenna **21** head and mesosoma, dorsal view **22** scape of antenna, dorso-lateral view **23** mesosoma, lateral view **24** head, mesosoma and first metasomal tergite, dorso-lateral view **25** fore tarsus **26** tip of ovipositor, lateral view **27** tip of ovipositor, dorsal view.

*Head* strongly transverse (Figs 10, 21). Vertex at least in anterior half densely rugose-reticulate with granulation. Eye with very short and rather dense setae. Ocelli arranged in obtuse triangle with base 1.1–1.3 times its sides. Occipital carina complete dorsally, archedly fused below with hypostomal carina weakly upper base of mandible. Frons with more or less distinct median carina in anterior half. Face weakly convex. Eye distinctly convergent below (Figs 14, 17). Tentorial pits deep, situated upper lower level of eyes. Malar suture distinct. Clypeal suture complete, but shallow. Mandible medium sized, almost not twisted. Palpi short, maxillary palpus 3-segmented, labial palpus 2-segmented. Antennae (Figs 11, 19, 20) weakly claviform, stable 17-segmented. Scape (Figs 12, 14, 17, 22) long, weakly curved, almost as long as maximum diameter of eye. Pedicel rather long, oval. First flagellar segment as long as or weakly longer than second segment. Segments in apical half of antenna weakly thickened. Apical segment pointed, but without spine.

*Mesosoma* (Figs 13, 16, 21, 23, 24). Pronope absent. Notauli complete, shallow and wide, densely rugulose-reticulate. Mesoscutum rugose-striate and setose on wide medioposterior half, smooth and glabrous on anterior and sublateral areas. Prescutellar depression deep and long. Scutellum convex, with lateral carinae, at least partly rugose. Sternaulus (precoxal sulcus) shallow, wide, long, densely rugulose. Prepectal carina distinct. Postpectal carina absent. Metapleural lobe long, wide, rounded apically. Propodeum strongly and abruptly rounded from median part (lateral view), weakly and widely longitudinally concave in medioposterior half (dorsal view), entirely rugulose-areolate, with lateral carinae.

*Wings* (Fig. 11, 15). Radial vein arising behind middle of pterostigma. Radial cell distinctly shortened. Second radial abscissa evenly and distinctly curved. First medial abscissa absent; as result, discoidal and first radiomedial cells fused. Mediocubital vein entirely sclerotised. Basal and recurrent veins distinctly convergent. Brachial cell short, widely open distally. Basal part of parallel vein weakly curved. In hind wing, first abscissa of mediocubital vein 4.0–5.0 times longer than second abscissa. Third abscissa of costal vein long; fourth abscissa almost straight.

*Legs* long and slender. Segments of median tarsus long. Fifth tarsal segments slender. Hind femur slender. Tarsal claws long, slender and weakly curved (Fig. 25).

*Metasoma* (Figs 11, 18). First tergite long, distinctly widened towards apex, its latero-ventral sides not fused, widely separated and with distinct split; dorsope small and very shallow; laterope indistinct. Second suture very shallow and fine. Only second tergite with separated laterotergites. Ovipositor (Figs 26, 27) long, straight, compressed laterally.

#### Distribution.

Western Palaearctic, Nearctic, Oriental Regions.

### Ecclitura primoris

Kokujev, 1902

http://species-id.net/wiki/Ecclitura_primoris

Figures 10 –27

#### Material examined.

Turkmenistan: female (holotype), “K.S. Anger”, “Askhabad, 8. VI. 98, on lamp”, “*Ecclitura Megaura primoris* ♀ Kok.” (ZISP). Italy: 4 females, Campania, Torre del Greco, (NA), MT, 18–30.VII.2007 (Guerrieri leg.); 7 females, Pisa, Ceppaiano Crespina, MT, 28.VI–12.VII, 12–24.VII, 24.VII–09.VIII, 04–20.IX, 20.IX–04.X.2012 (A. Loni leg.); 7 females, Pisa, Poggio a Casone Crespina, MT, 12–24.VII, 24.VII–09.VIII, 09.VIII–04.IX, 20.IX–04.X.2012 (A. Loni leg.). Turkey: 1 female, “TR Edirne, Trakya, Univ. Biyoloji Bol. (Noct.) 16.9.1999”

#### Redescription.

**Female**. Body length 2.3–2.5 mm; fore wing length 1.9–2.2 mm.

*Head*. Width of head 2.1–2.3 times its median length, 1.4–1.5 times width of mesoscutum. Head behind eye strongly and weakly-curvedly narrowed. Length of eye (dorsal view) 1.8–2.0 times longer than temple. POL 1.0–1.5 times Od, 0.3–0.4 times OOL. Minimum width of face 0.70–0.75 times its height, 0.5–0.6 times its maximum width at level of antennal toruli or level of upper margins of eyes, almost equal to width of clypeus, about 0.9 times minimum (transverse) diameter of eye. Maximum diameter of eye 1.5–1.6 times its minimum diameter. Malar space very short, 0.10–0.13 times maximum diameter of eye, 0.4–0.5 times basal width of mandible. Distance between tentorial pits 2.5–3.0 times distance from pit to eye. Width of cly-peus 1.9–2.0 times its median height.

*Antennae* 17-segmented, weakly claviform. Scape 1.0–1.2 times as long as height of face, 5.5–6.5 times longer than maximum width anteriorly, 4.0–5.0 times longer than maximum width laterally. Pedicel 1.3–1.5 times longer than wide. First flagellar segment 4.0–5.0 times longer than its maximum apical width, about as long as second segment. Penultimate segment 1.5–1.8 times longer than wide, 0.50–0.55 times as long as first segment, 0.5–0.8 times as long as apical segment.

*Mesosoma*. Length 1.4–1.5 times its median width. Mesoscutum highly and subvertically elevated above pronotum. Scutellum distinctly convex. Prescutellar depression long, with high median and often two to four lateral carinae, finely rugulose between carinae, depression about 0.3 times as long as scutellum. Scutellum 1.1–1.3 times longer than its anterior width. Subalar depression shallow, rather wide, densely rugulose-reticulate. Sternaulus complete, shallow, curved, coarsely rugulose-reticulate. Posterior mesopleural furrow (along mesopleural suture) coarsely crenulate.

*Wings*. Length of fore wing 2.7–2.8 times its width. Metacarp (inside radial cell) 0.7–0.9 times as long as pterostigma, 2.0–2.4 times longer than distance between apex of radial cell and apex of wing. Radial cell 2.4–2.5 times longer than maximum width. First radial abscissa about 0.5 times as long as maximum width of pterostigma. First radiomedial vein weakly sinuate or straight, 2.5–3.0 times longer than first radial abscissa, 0.3–0.4 times as long as second radial abscissa, 0.7–0.9 times as long as recurrent and second abscissa of medial veins combined. Distance between nervulus and basal vein 0.5–0.8 times nervulus length. Parallel vein basally sclerotised and weakly curved, unsclerotised its most apical part. Hind wing 3.8–4.1 times longer than maximum width.

*Legs*. Hind femur 4.9–5.5 times longer than maximum width. Hind tarsus almost as long as hind tibia. Hind basitarsus 0.6–0.7 times as long as second-fifth segments combined. Second segment of hind tarsus 0.45–0.50 times as long as basitarsus, 1.15–1.25 times longer than fifth segment.

*Metasoma*. First tergite almost regularly and distinctly widened from base to apex, with fine spiracular tubercle in basal third. Apical width of first tergite 2.3–2.8 times its minimum width; length of tergite 1.8–2.0 times its apical width. Length of second and third tergites combined 1.1–1.3 times basal width of second tergite, 0.9 times their maximum width. Ovipositor sheath 0.6–0.7 times as long as metasoma, 1.4–1.5 times longer than first tergite, 0.7–0.8 times as long as mesosoma, and 0.30–0.35 times as long as fore wing.

*Sculpture and pubescence*. Head mainly and densely rugose-reticulate with granulation, vertex sometimes almost smooth posteriorly. Mesoscutum smooth in anterior half of median lobe and on wide median areas of lateral lobes, rugose-reticulate wide along notauli and rugose-striate on wide area in medioposterior half. Scutellum almost entirely distinctly rugose-reticulate, partly with granulation, sometimes smooth or almost smooth on medio-anterior third or anterior half. Mesopleuron mainly reticulate-rugulose, smooth or almost smooth medially on rather small area. Propodeum without areas, coarsely and rather densely reticulate-areolate, with fine rugulosity. Hind femur densely and rather distinctly rugulose-granulate. First metasomal tergite densely striate in basal 0.6–0.8, smooth in apical 0.2–0.4. Remaining tergites entirely smooth. Mesoscutum glabrous on smooth areas, coved by short setae on sculptured areas. Face densely and very shortly setose.

*Colour*. Body mainly brownish yellow or yellow, metasoma often almost entirely yellow. Antennae yellow, infuscate in apical half. Palpi yellow. Legs yellow, all legs brownish. Ovipositor sheath almost black. Wings hyaline. Pterostigma entirely yellow.

**Male**. Unknown.

#### Distribution.

Tajikistan, Turkmenistan, Iran, Azerbaijan, Russia (Dagestan), Turkey (first record), Albania, Italy (first record).

## Supplementary Material

XML Treatment for Histeromerus

XML Treatment for Histeromerus mystacinus

XML Treatment for Ecclitura

XML Treatment for Ecclitura primoris
